# Meeting of the Medical Association of Georgia

**Published:** 1881-05

**Authors:** 


SECRETARY'S OFFICE, MEDICAL ASSOCIATION OF GA.,
No 112 McIntosh Street,
AUGUSTA, GA., MAY 7TH, 1881.

The Thirty-second Annual Session of the Medical Association of Georgia, was held in Thomasville, on April 20th and 21st, 1881. The following are the officers for the ensuing year:—President, William F. Holt, Macon; First Vice-President, Eugene Foster, Augusta; Second Vice-President, T. M. McIntosh, Thomasville; Secretary, A. Sibley Cambell, Augusta; Treasurer, K. P. Moore, Forsyth.

The next session will be held in Atlanta, on the third Wednesday in April (19th), 1882.

Communications should be addressed to the undersigned.

A. SIBLEY CAMPBELL, Secretary.

We would be obliged to Dr. Campbell for any interesting paper or papers, read before the Association, for publication in the INDEPENDENT PRACTITIONER.—Ed.

